# YesU from *Bacillus subtilis* preferentially binds fucosylated glycans

**DOI:** 10.1038/s41598-018-31241-8

**Published:** 2018-09-03

**Authors:** Joe Tiralongo, Oren Cooper, Tom Litfin, Yuedong Yang, Rebecca King, Jian Zhan, Huiying Zhao, Nicolai Bovin, Christopher J. Day, Yaoqi Zhou

**Affiliations:** 1 Institute for Glycomics, Griffith University, Gold Coast Campus, QLD 4222, Australia; 2 School of Data and Computer Science, Sun Yat-Sen University, Guangzhou, People’s Republic of China; 3 Queensland Institute of Medical Research, Brisbane, Queensland Australia; 4 Shemyakin Institute of Bioorganic Chemistry, Russian Academy of Sciences, Moscow, Russia

## Abstract

The interaction of carbohydrate-binding proteins (CBPs) with their corresponding glycan ligands is challenging to study both experimentally and computationally. This is in part due to their low binding affinity, high flexibility, and the lack of a linear sequence in carbohydrates, as exists in nucleic acids and proteins. We recently described a function-prediction technique called SPOT-Struc that identifies CBPs by global structural alignment and binding-affinity prediction. Here we experimentally determined the carbohydrate specificity and binding affinity of YesU (RCSB PDB ID: 1oq1), an uncharacterized protein from *Bacillus subtilis* that SPOT-Struc predicted would bind high mannose-type glycans. Glycan array analyses however revealed glycan binding patterns similar to those exhibited by fucose (Fuc)-binding lectins, with SPR analysis revealing high affinity binding to Lewis^x^ and lacto-*N*-fucopentaose III. Structure based alignment of YesU revealed high similarity to the legume lectins UEA-I and GS-IV, and docking of Lewis^x^ into YesU revealed a complex structure model with predicted binding affinity of −4.3 kcal/mol. Moreover the adherence of *B. subtilis* to intestinal cells was significantly inhibited by Le^x^ and Le^y^ but by not non-fucosylated glycans, suggesting the interaction of YesU to fucosylated glycans may be involved in the adhesion of *B. subtilis* to the gastrointestinal tract of mammals.

## Introduction

To date many classes of carbohydrate binding proteins (CBPs) have been identified, including lectins such as F-type^1^, C-type^2^, and Galectins^3^, and carbohydrate binding modules (CBMs) associated with glycoside hydrolases or glycosidases^4^ that occur ubiquitously in nature. Lectins regulate numerous crucial biological processes including pattern recognition of pathogens, correct folding of glycoproteins^5^ and cell-cell communication^6^. Lectin-oligosaccharide interactions are highly specific due to the branching motifs of either homo- or hereteopolymers of monosaccharide units allowing for structural complexity^7,8^ that are crucial determinants for many biological interactions at a cellular level. Over 50% of all newly synthesized proteins and lipids are glycosylated^9^, resulting in cell surface decoration of glycoproteins and glycolipids that are known to play central roles in cell development, tumour progression and metastasis^10,11^.

Intensive research aimed at recognizing altered cell surface glycosylation during disease development using CBPs is becoming increasingly important for both biomarker discovery and inhibitor design. As a result it is essential that CBPs encoded within any given genome be identified and their carbohydrate specificity determined. Employing computational function prediction to guide experimental analysis has the potential to significantly aid in this endeavour.

The first generation techniques used to automate protein function annotation was based on sequence homology. Modern approaches now employ machine-learning classifiers based on the protein evolutionary data, structure geometric, or other sequence or structure information^12^. Recently, we introduced a template-based method (SPOT-Struc) that predicts CBPs by making structural alignment between a query structure and the template structure of a known CBP. Highly aligned structures are followed by a binding affinity prediction to further remove potentially false positive CBPs. Using this method we predicted several structural genome targets as CBPs, one of which was YesU from *Bacillus subtilis* (RCSB Protein Data Bank code 1oq1)^13^.

YesU, even after a decade of genome engineering^14^, remains an uncharacterized protein with unknown function. In 2005, Structural Classification of Proteins–extended (SCOPe) classed YesU as a beta protein with a galectin-like fold, being a member of the concanavalin-A (ConA) like lectins/glucanses superfamily^15^ possessing a β-sandwich structure comprising 12–14 strands organised as 2 sheets to form a jellyroll topology^16^. Our SPOT-Struc analysis specifically matched YesU to the integral membrane mammalian protein VIP36 (2e6v)^13^, a known leguminous type lectin with a β-sandwich and jellyroll fold^17,18^ that recognizes high Man-type glycans^19^.

Here we describe the experimental verification of YesU as a CBP, and its functional annotation as a new Lewis^x^ (Le^x^) binding lectin using a combination of glycan array profiling followed by detailed affinity analysis using surface plasmon resonance (SPR). Our data demonstrates the complementary role of computational prediction and experimental validation in function annotation.

## Results

### Expression of Recombinant YesU and Glycan Array Analysis

**Figure 1:**
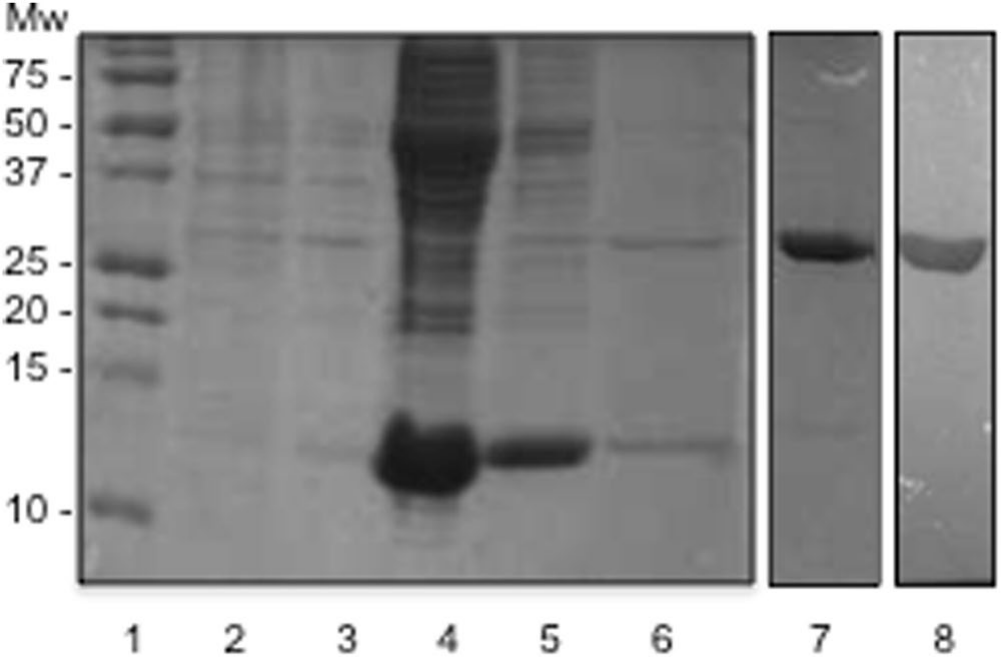
Coomassie stained gel and subsequent western blot of the expression and purification of *B. subtilis* hypothetical cytosolic protein 031524 (YesU). Lane 1: Biorad precision plus protein standard, Lane 2: uninduced culture, Lane 3: induced culture, Lane 4: slurry mix (protein lysate and resin), Lane 5 wash ^#^1 (binding buffer), Lane 6: wash ^#^2 (wash buffer), Lane 7: concentrated purified YesU protein (220 residues) concentrated using Amicon 3 K 15 mL centrifugal filter (2 mg/mL), Lane 8: Subsequent western blot of purified YesU protein (220 residues) concentrated using Amicon 3 K 15 mL centrifugal filter at the expected 25.2 kDa.

**Figure 2:**
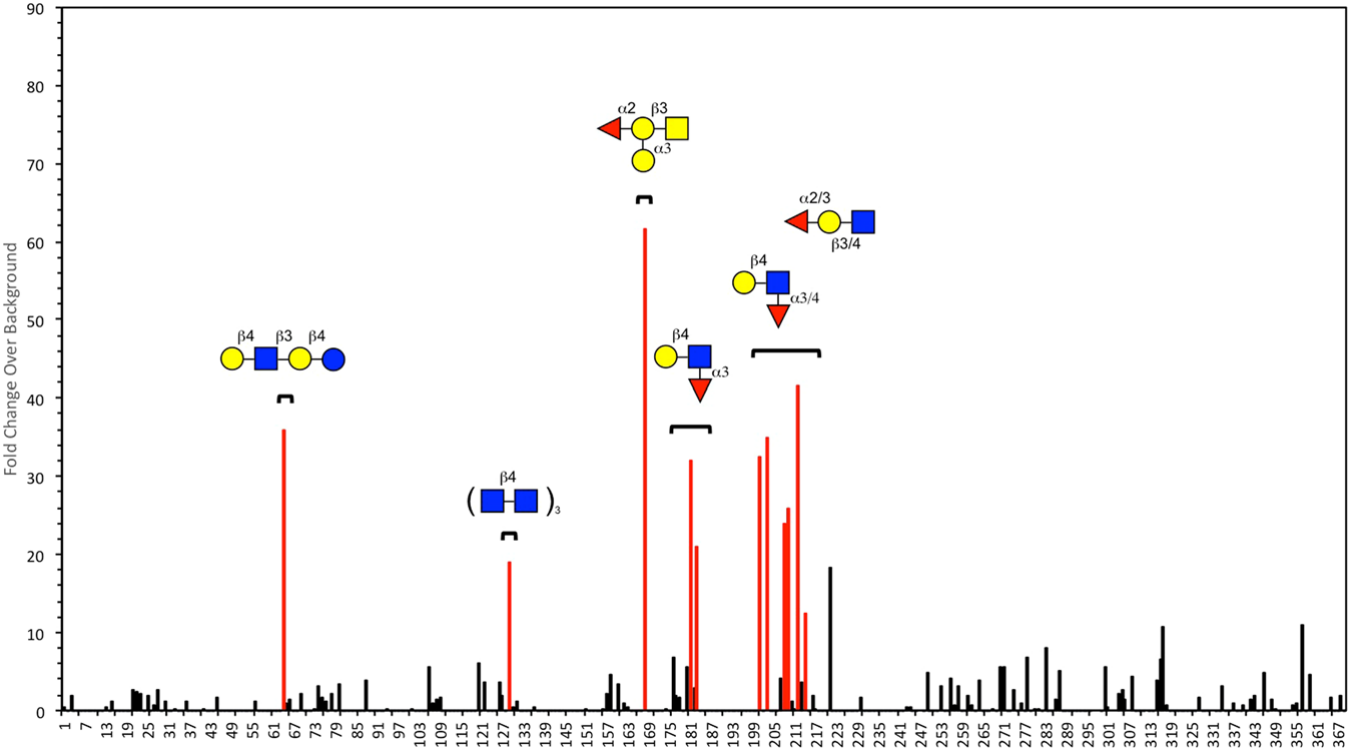
Glycan array analysis of YesU. Binding of YesU to glycan structures present on the array was assessed both for statistical significance using T-tests (p < 0.05, red bars), and by determining fold-change over background. Black bars indicated statistical insignificant (p > 0.05) fold-change over background. The Symbol Nomenclature for Glycans (SNFG)^55^ was used to represent glycan structures (yellow circle, Gal; blue square, GlcNAc; yellow square, GalNAc; red triangle, Fuc). The full list of glycans present on our array and their corresponding glycan ID and structure is provided in Table S1.

Recombinant *B. subtilis* hypothetical cytosolic protein 031524 (YesU) was expressed in *E. coli* BL21 (DE3) cells using the vector pMCSG68 obtained from DNASU (Arizona State University), and purified to homogeneity. Figure 1 illustrates the successful purification of highly pure His-tagged YesU (220 residues, 25.2 kDa) using His-select nickel affinity resin (lane 7 coomassie stain and lane 8 Western blot) that was used in subsequent analyses. Size exclusion chromatography showed the YesU in solution is predominantly monomeric with a molecular mass of approximately 25 kDa (Fig. S1), which is consistent with its structural annotation as a monomeric protein. The glycan binding potential and specificity of recombinant YesU was initially assessed using glycan array analysis, a now well-established and widely utilised tool for high-throughput screening of CBP specificity, and whose relative densities are comparable to that seen in a biological context^20,21^. Interactions were verified through both visual inspection of scanned images and statistical analysis (P < 0.05). The evaluation of YesU by glycan array analysis revealed a limited glycan binding profile, with YesU exhibiting statistically significant binding only to fucosylated glycans (Fig. 2). Importantly, YesU displayed no significant binding to any Man structures present on the array, even though VIP36, the lectin matched to YesU using SPOT-Struc, exclusively binds high-Man type glycans^17^. Of the fucosylated structures bound, significant binding was observed to fucosylated glycans that share a lacto-*N*-neotetraose (LNnT) backbone and at least one non-terminal α1-3 fucose (Fuc) (8C, 8H, 8I, 8L, 8N), and to Lewis (8A, 539, 542) and Blood Group B (363) structures (Fig. 2 and Table S1). Interestingly, similar but truncated fucosylated LNnT structures (7A–7E) were not bound by YesU under the experimental glycan array conditions used. Grant *et al*., recently reported the potential of false-negative binding on glycan arrays arising from restricted glycan presentation^22^. Therefore in subsequent SPR experiments undertaken to verify and further characterize YesU’s carbohydrate binding specificity, truncated fucosylated LNnT structures were also evaluated. In addition to binding terminal and non-terminal Fuc-containing glycans, interaction with LnNT (383) and *N,N′*, *N″*, *N″′*, *N″″*, *N″″′*-Hexaacetyl chitohexaose ((GlcNAc)_6_, 4D) was also observed (Fig. 2), and as such were also assessed by SPR.

### Surface Plasmon Resonance Analysis

Glycan array analysis strongly suggested that YesU recognises terminal and non-terminal Fuc-containing glycans. To further quantitatively explore the interaction of YesU with these glycans SPR was performed. SPR analysis revealed preferential binding to Lewis type, Blood Group type and fucosylated LNnT glycans, in particular non-terminal Fuc variants (Table 1 and Fig. S3). A comparison of YesU’s binding to Lewis and related structures demonstrates an increased affinity for non-terminal α1-2/3-Fuc, revealed through a 3-fold greater affinity for Lewis^x^ (Le^x^, 7I, *K*_D_: 0.15 ± 0.006 μM), lacto-*N*-fucopentaose III (LNFP III, 7 C: *K*_D_: 0.14 ± 0.001 μM) and Blood group B trisaccharide (BGBT, 7 M, *K*_D_: 0.19 ± 0.003 μM) over Lewis^a^ (Le^a^, 7 J, *K*_D_: 0.42 ± 0.004 μM) and lacto-*N*-difucohexaose II (LNDFH II, 7E: *K*_D_: 0.34 ± 0.01 μM) that both possess a non-terminal α1-4 branched Fuc. Similarly, monofucosyl(1-3)-iso-lacto-*N*-octaose (MFiLNO, 8 N, *K*_D_: 0.24 ± 0.02 μM) also showed higher affinity over glycans with terminal Fuc, although with a slight reduction in *K*_D_ probably due to the presence of a terminal lacto-*N*-biose I (Galβ1-3GlcNAc). The observed decrease in affinity for Lewis^y^ (Le^y^, 7 N, *K*_D_: 0.34 ± 0.002 μM) could be due to the presence of a terminal α1-2-Fuc. This is supported by the observation that the structurally related histo-blood group antigens ABH(O); H-disaccharide (HDi, 7 F, *K*_D_: 0.36 ± 0.01 μM) and Blood Group H type II trisaccharide (BGHT II, 7 O, *K*_D_: 0.34 ± 0.004 μM) exhibited a 2–3-fold lower affinity. Similarly, 8 H (LNnDFH I), 7 A (LNFP I), and 7 K (BGAT) displayed a reduced affinity compared to saccharides with only a non-terminal α1-3-Fuc residue. Further the absence of Fuc as in 1 H (LnNT, Galβ1-4GlcNAcβ1-3Galβ1-4Glc), or a charged group as in 8 A (sulpho-Lewis^a^) and 10B (sialyl-Lewis^x^) to the core Lewis type structure lead to a reduction or complete loss of binding. Glycan array analysis also revealed potential binding of YesU to *N*,*N′*,*N″*,*N″′*,*N″″*,*N″″′*-Hexaacetyl chitohexaose ((GlcNAc)_6_, 4D). Although this interaction was verified by SPR, it was of significantly weaker affinity (*K*_D_: 1.13 ± 0.02 μM) compared to Le^x^ and LNFP III (Table 1). As was also observed by glycan array analysis, no interaction of YesU with α1-2- and α1-6-mannobiose (5 C, 5 F), α1-3-galactobiose (1N), and LNT (1G) was detected by SPR. Taken together, SPR analysis clearly highlights the importance of a non-terminal Fuc and a lesser extent terminal Fuc for glycan binding by YesU.

**Table 1:** Dissociation constants (K_D_) of YesU (50 μg/mL) His tagged to NTA chip for selected glycans.

ID	Name	Structure	K_D_ (μM)
7C	LNFP III	Galβ1-4(Fucα1-3)GlcNAcβ1-3Galβ1-4Glc	0.14 ± 0.001
7I	Le^x^	Galβ1-4(Fucα1-3)GlcNAc	0.15 ± 0.006
7M	BGBT	Galα1-3(Fucα1-2)Gal	0.19 ± 0.003
8N	MFiLNO	Galβ1-3GlcNAcβ1-3Galβ1-4(Fucα1-3)GlcNAcβ1-6(Galβ1-3 GlcNAcβ1-3)Galβ1-4Glc	0.24 ± 0.02
7N	Le^y^	Fucα1-2Galβ1-4(Fucα1-3)GlcNAc	0.34 ± 0.002
7O	BGHT II	Fucα1-2Galβ1-4GlcNAc	0.34 ± 0.004
7E	LNDFH II	Galβ1-3(Fucα1-4)GlcNAcβ1-3Galβ1-4(Fucα1-3)Glc	0.34 ± 0.01
7F	HDi	Fucα1-2Gal	0.36 ± 0.01
7J	Le^a^	Galβ1-3(Fucα1-4)GlcNAc	0.42 ± 0.004
1H	LNnT	Galβ1-4GlcNAcβ1-3Galβ1-4Glc	0.44 ± 0.02
4D	(GlcNAc)_6_	GlcNAcβ1-4GlcNAcβ1-4GlcNAcβ1-4GlcNAcβ1-4GlcNAcβ 1-4GlcNAc	1.13 ± 0.02
8H	LNnDFH I	Fucα1-2Galβ1-4(Fucα1-3)GlcNAcβ1-3Galβ1-4Glc	1.14 ± 0.03
7A	LNFP I	Fucα1-2Galβ1-3GlcNAcβ1-3Galβ1-4Glc	1.23 ± 0.02
8A	Sulfo-Le^a^	SO_3_-3Galβ1-3(Fucα1-4)GlcNAc	2.07 ± 0.02
7K	BGAT	GalNAcα1-3(Fucα1-2)Gal	7.63 ± 0.30
1N	α1-3-Galactobiose	Galα1-3Gal	—
10B	Sialyl-Le^x^	Neu5Acα2-3Galβ1-4(Fucα1-3)GlcNAc	—
5C	α1-2-Mannobiose	Manα1-2Man	—
5F	α1-6-Mannobiose	Manα1-6Man	—
1G	LNT	Galβ1-3GlcNAcβ1-3Galβ1-4Glc	—

Glycans were tested in triplicate (FC 2/3/4). FC 1 was used as a reference blank to calculate mean K_D_ and SD from each flow cell. Chi Square variance is less than 10% for all calculated affinities. Sensorgrams and plots of response at equilibrium against concentration for each glycan for which K_D_’s were determined are given in Fig. S3.

LNFP III, Lacto-*N*-fucopentaose III; BGBT, Blood Group B Trisaccharide; BGHT II, Blood Group H Type II trisaccharide; HDi, H-disaccharide; LNDFH II, Lacto-*N*-difucohexaose II; MFiLNO, Monofucosyl(1-3)-iso-lacto-*N*-octaose; LNnDFH I, Lacto-*N*-neodifucohexaose I; (GlcNAc)_6_, N,N′,N″,N″′,N″″,N″″′-Hexaacetyl chitohexaose; LNFP I, Lacto-*N*-fucopentaose I; BGAT, Blood Group A trisaccharide; LNnT, Lacto-*N*-neotetraose; –, No concentration dependent binding.

As previously mentioned large fucosylated glycans that share an LNnT backbone and at least one non-terminal α1-3 Fuc (eg. 8C, 8H, 8I, 8L, 8N) were bound by YesU on our glycan array but the corresponding truncated fucosylated LNnT structures (7A-7E) were not. Quantitative analysis by SPR verified the interaction of YesU with monofucosyl(1-3)-iso-lacto-*N*-octaose (MFiLNO, 8 N: *K*_D_: 0.24 ± 0.02 μM) and lacto-*N*-neodifucohexaose I (LNnDFH I, 8 H: *K*_D_: 1.14 ± 0.03 μM), further highlighting the need to consider restricted glycan presentation when assessing glycan array data^22^. Despite this, glycan array analysis successfully predicted the glycan binding specificity of YesU that was subsequently verified and refined by SPR.

### Structure-based alignment and Le^x^ docking analysis

**Figure 3:**
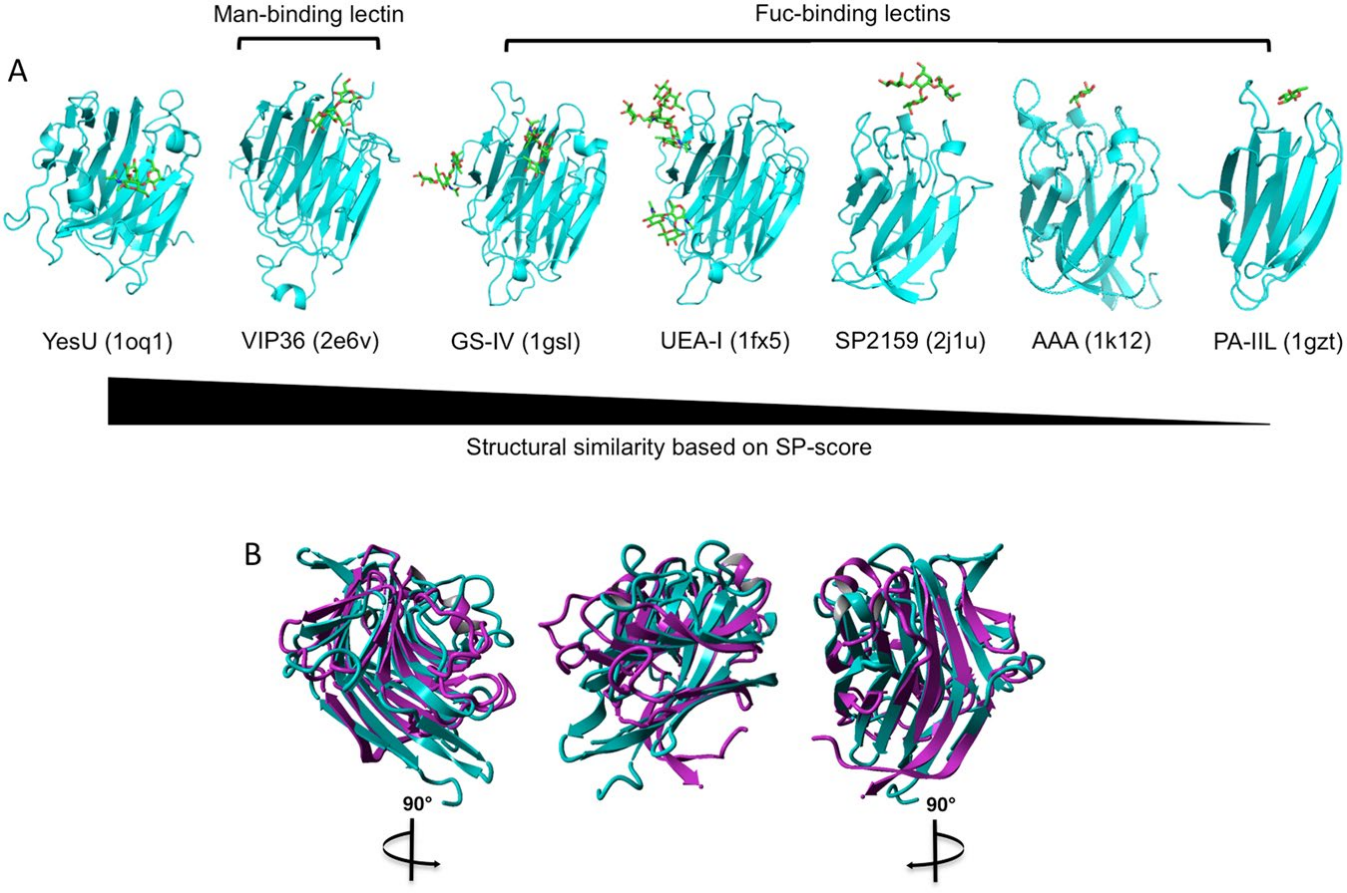
Side-by-side representation of YesU with five Fuc-binding lectins (GS-IV, pdbID: 1gsl; UEA-I, pdbID: 1fx5; SP2159 pdbID: 2j1u; AAA, pdbID: 1k12; PA-IIL, pdbID: 1gzt) and VIP36 (pdbid: 2e6v) that were used for structure-based alignment by SPalign. All bound ligands shown (with the exception of YesU (1oq1)) are those associated with the corresponding PDB entries (**A**). YesU (magenta) aligned to the structure of GS-IV (cyan) by SPalign (**B**).

**Figure 4:**
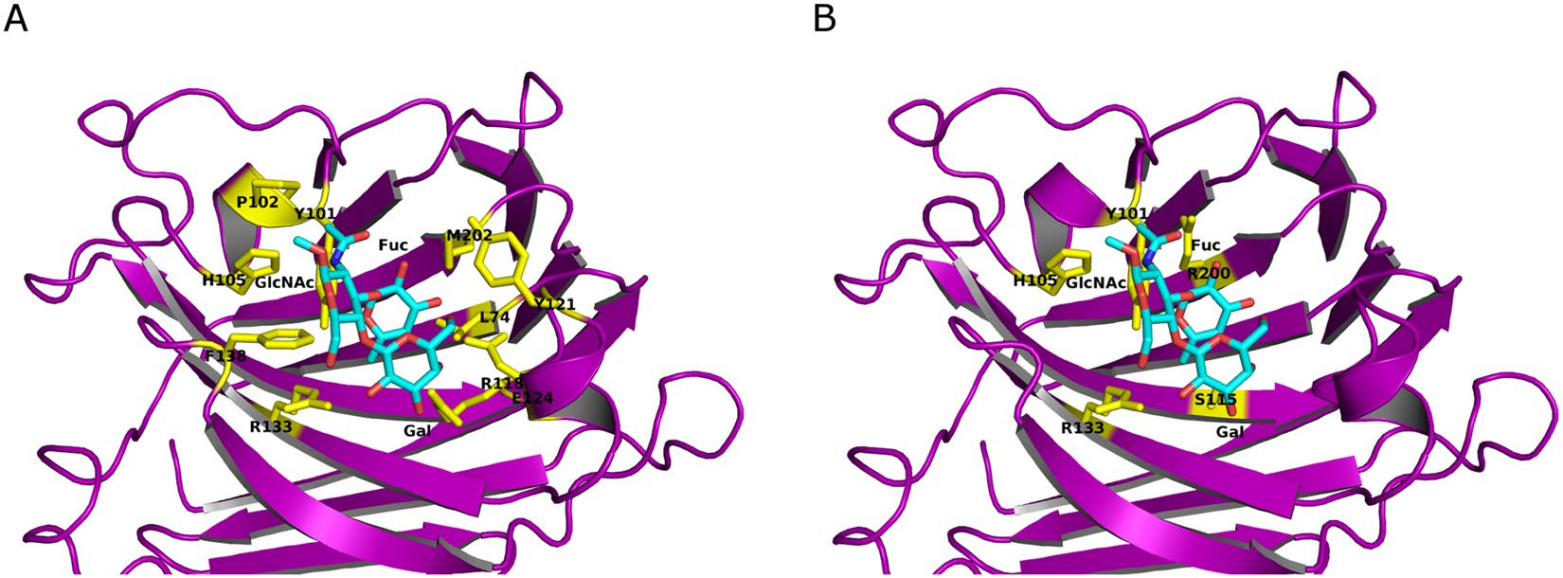
The docking of Le^x^ into YesU using Vina-Carb with default parameters revealed a potential Le^x^ binding orientation in the final complex structure model with predicted binding affinity of −4.3 kcal/mol. Predicted interacting residues are highlighted in yellow (**A**). The YesU-Le^x^ binding site was also independently predicted by the purely sequence-based technique SPRINT-CBH. Predicted binding residues within 7 Å of the docked Le^x^ are highlighted in yellow (**B**).

Glycan array and SPR analyses confirmed the prediction that YesU is a carbohydrate binding protein. However, it was expected that YesU would display a similar Man-binding profile to that described for VIP36, the template that was best matched structurally to YesU (SP-score of 0.80, Table 2). To better understand the discrepancy between the YesU predicted and observed carbohydrate binding specificity, we structurally compared YesU to five Fuc-binding lectins (Table 2 and Fig. 3A), lectin II from *Pseudomonas aeruginosa* (PA-IIL, pdbID: 1gzt), the *Anguilla anguilla* agglutinin (AAA, pdbID: 1k12), SP2159 from *Streptococcus pneumoniae* (pdbID: 2j1u), lectin I from *Ulex europaetus* (UEA-I, pdbID: 1fx5), and lectin IV from *Griffonia simplicifolia* (GS-IV, pdbID: 1gsl), by SPalign^23^. This analysis revealed that YesU is more structurally similar to the legume lectins UEA-I and GS-IV than it is to the bacterial lectins PA-IIL and SP2159. Importantly, YesU’s similarity to UEA-1 and GS-IV is comparable to that observed with VIP36. As shown in Table 2, in total 185 and 177 amino acids in YesU and UEA-I and GS-IV were aligned with an average root-mean-squared distance of 2.86 Å (SP-score of 0.75) and 2.68 Å (SP-score of 0.75), respectively. That is, global structural similarity is high enough for function transfer. The structural alignment of YesU and GS-IV is depicted in Fig. 3B, and shows that even though these lectins have a sequence identity of only 13.7% (Table 2), there is a high degree of structural similarity; with key features of the conventional legume lectin fold^24,25^ being conserved in YesU. Based on the structural alignments in Fig. 3, there is no clear consensus binding site for Le^x^ in YesU. GS-IV was selected as a representative binding template due to its fucosylated glycan binding characteristics, high structural similarity with YesU, and the proximity of its binding site to the VIP36 Man-type glycan binding site. A potential Le^x^-YesU binding site was identified based on a structural alignment with the Le^b^-GS-IV binding complex (Table 2 and Fig. 3). Docking was subsequently performed by Vina-Carb in a 30 Å box around the predicted site. This docking experiment revealed a potential Le^x^ binding orientation, with predicted binding affinity of −4.3 kcal/mol (Fig. 4A). While the minimised conformation was translated somewhat away from the original, predicted binding site based on the template of the Le^b^-GS-IV binding complex (Fig. 3A), the docked conformation is consistent with the site independently predicted by a purely sequence-based technique SPRINT-CBH^26^. Of the top 10 predicted binding residues by SPRINT-CBH, Arg200, His105, Arg133, Tyr101 and Ser115 are all within 7 Å of the docked Le^x^ conformation (Fig. 4B), with His105, Arg133 and Tyr101 also predicted by Vina-Carb to interact with Le^x^ (Fig. 4A). While Arg125, Lys196, Trp51, Ser40 and Tyr158 are also predicted as potential binding residues by SPRINT-CBH, they are largely isolated on the protein surface and are unlikely to form part of the true binding site. Geometric clustering has previously been used to strengthen the signal/noise ratio of structure-based, functional annotation tools. In this case, the geometric distribution of predicted binding residues supports the binding site selected by the docking experiment.

**Table 2:** Structure based alignment of YesU to five Fuc-binding lectins using SP-align.

Lectin	Ligand	pdbID	SP-score	Nali*	% alignment	RMSD	% sequence identity
VIP36	Manα1-2Manα1-2Man High Man type)	2e6v	0.80	183	82.0%	2.46	15.5%
UEA-I	Fucα1-2Galβ1-4GlcNAc (BGHT II)	1fx5	0.75	185	82.9%	2.86	15.2%
GS-IV	Fucα1-2Galβ1-3/4(Fucα1-4/3) GlcNAc (Le^b^/Le^y^)	1gsl	0.75	177	79.4%	2.68	13.7%
SP2159	Fucα1-2Galβ1-4(Fucα1-3) GlcNAc (Le^y^)	2j1u	0.52	113	50.7%	2.97	12.2%
AAA	Galβ1-3(Fucα1-4)GlcNAc (Le^a^)	1k12	0.50	120	53.8%	3.18	17.4%
PA-IIL	Galβ1-3(Fucα1-4)GlcNAc (Le^a^)	1gzt	0.39	79	35.4%	3.06	13.8%

*Nali, Number of aligned residue pairs; RMSD, Average root-mean-squared distance.

### *B. subtilis* adherence to intestinal cells is inhibited by fucosylated glycans

**Figure 5:**
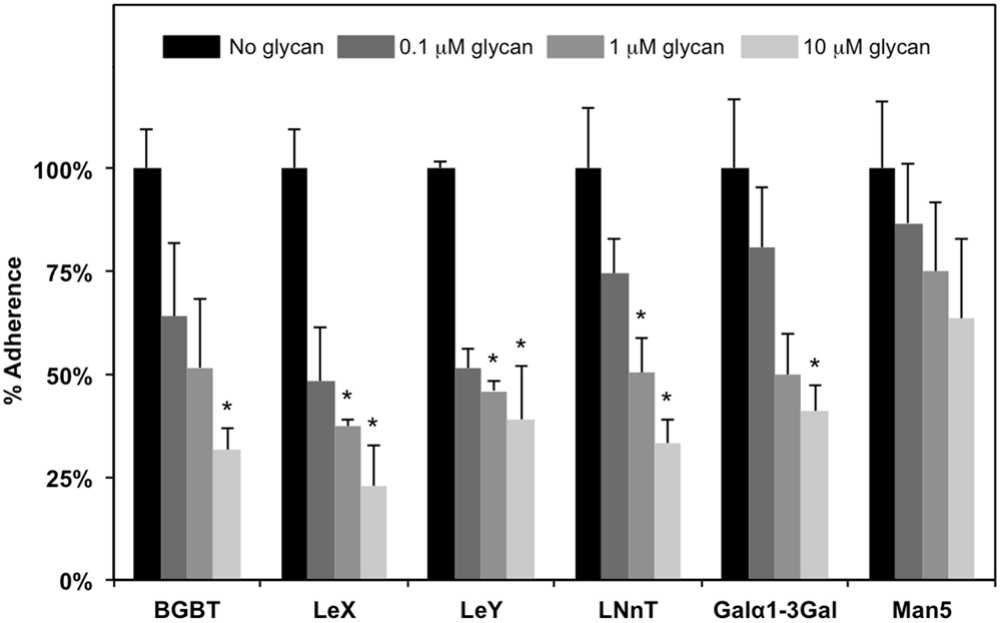
The adherence of *B. subtilis* to the intestinal cell line Caco-2 was inhibited by a range of fucosylated glycans including BGBT, Le^x^, and Le^y^, as well as by LNnT but to a slightly lesser extent. No significant inhibition of *B. subtilis* to Caco-2 cells was observed for the high Man-type glycan, Manα1-3(Manα1-3(Manα1-6)Manα1-6)Man (Man5). *Significant difference to control containing no glycan, P < 0.05.

Our data suggests that YesU from *B. subtilis* preferentially binds fucosylated glycans such as Le^x^. Fucosylated glycans, particularly Lewis blood group antigens are known to play a functional role in the adhesion of a number of bacteria to the gastrointestinal tract. In order to explore the possibility that YesU has a similar function in *B. subtilis* we investigated the ability of fucosylated and non-fucosylated glycans to inhibit the adherence of *B. subtilis ATCC6633* (expression of YesU in *B. subtilis ATCC6633* was confirmed by RT-PCR, data not shown) to the intestinal cell line Caco-2. Figure 5 shows that Le^x^ and Le^y^ at 1 μM were able to significantly inhibit *B. subtilis* adherence by greater than 50%, whereas the high Man-type glycan, Manα1-3(Manα1-3(Manα1-6)Manα1-6)Man (Man5) showed no significant inhibition at concentrations up to 10 μM, and Galα1-3 Gal and BGBT (Galα1-3(Fucα1-2)Gal) only exhibited significant inhibition at 10 μM (approximately 50% at 10 μM). LNnT (Galβ1-4GlcNAcβ1-3Galβ1-4Glc) was also inhibitory but not to the same extent as Le^x^ and Le^y^, which mimics the SPR data that showed preferential binding of YesU to Le^x^ and Le^y^ over BGBT and LNnT.

## Discussion

YesU is located between a rhamnogalacturonan acetylesterase (RhgT) and a DUF624 domain containing protein in the *B. subtilis* genome. The location of YesU between these two genes is fairly well conserved with 20 complete *B. subtilis* genomes containing the identical alignment and an additional 32 complete genomes containing the same up and downstream genes around YesU, with overlap between the gene start and finish codons for YesU and the downstream DUF624 protein. YesU in all genomes assessed had its own promoter (BPPROM^27^) with the upstream RhgT gene possessing a transcription terminator (using RNAmotif^28^ and Erpin^29^).

We had previously predicted, using SPOT-struc, YesU to be a carbohydrate binding protein^13^ with specificity similar to the Man-binding protein VIP36 (pdbID: 2e6v)^17,18^. VIP36 exhibits a typical β-sandwich and jellyroll fold^17,18^ that is a common structural characteristic of the ConA-like lectin/glucanse superfamily^15^. However, wide scale glycan array screening of YesU did not display any binding to terminal mannosyl structures or even oligosaccharides containing sub-terminal Man. This lack of binding was validated by SPR with no binding observed to both α1-2-mannobiose and α1-6-mannobiose structures. Our glycan array and SPR analysis instead suggests that YesU’s carbohydrate specificity is more closely related to that of Fuc-binding lectins than Man-binding lectins.

Fuc-binding lectins have been identified in a range of organisms from bacteria to vertebrates. Interestingly, similar to VIP36 and other members of the ConA-like lectin/glucanse superfamily, F-type lectins (fucolectins) also possess a β-barrel with jellyroll topology that defines a typical F-type fold. Two of the best-characterized F-type lectins, both structurally and biochemically, are AAA (*Anguilla anguilla* agglutinin) from the European eel, and SP2159 from the Gram-positive pathogen *Streptococcus pneumoniae*. AAA binds terminal Fuc residues in certain blood group antigens, including H type 1 (Fucα1-2Galβ1-4GlcNAc) and Le^a^ (Galβ1-3(Fucα1-4)GlcNAc)^30^, while SP2159 binds to a more distinct set of fucosylated oligosaccharides compared to AAA, including the H, A and B blood group antigens and Le^y^ (Fucα1-2Galβ1-4(Fucα1-3)GlcNAc) epitope, but not Le^x^ (Galβ1-4(Fucα1-3)GlcNAc) or Le^a^ Galβ1-3(Fucα1-4)GlcNAc)^31,32^. The glycan binding profile of AAA^33^, and the glycan binding domain CBM47^31^ of SP2159^34^ (also referred to as *Sp*GH98), have also been analysed by glycan array through the Consortium for Functional Glycomics (CFG). Interestingly, even though both lectins did bind Fuc, they only bound a very restricted subset of fucosylated glycans on the CFG array. AAA, analysed on the CFG glycan array version 2.1, only bound Fucα1-2GlcNAcβ-Sp8 and Fucα1-3GlcNAcβ-Sp8 with any significance, and SP2159, analysed on CFG glycan array version 3.0, only bound Lewis^y^ (Fucα1-2Galβ1-4(Fucα1-3)GlcNAcβ-Sp2), 2′-fucosylactose (Fucα1-2Galβ1-4Glcβ–Sp1) and Lewis^b^ (Fucα1-2Galβ1-3(Fucα1-4)GlcNAcβ-Sp2), with any significance. The narrow Fuc-binding profile observed for AAA and SP2159 is similar to that observed for YesU on our glycan array, and as such may also reflect the influence of restricted glycan presentation on the CFG glycan array^22^.

Further examples of Fuc-binding lectins include UEA-1 and GS-IV from the plants *Ulex europaeus* and *Griffonia simplicifolia*, respectively. GS-IV is known to bind Le^b^ and Le^y^ with high affinity^25^, and UEA-I interacts strongly with α1,2 linked Fuc specifically H type 2 trisaccharides^24^. CFG Glycan array data is only available for UEA-I^35^ with specific binding on the CFG glycan array version 4.0 observed to blood group H type saccharides possessing the terminal disaccharide Fucα1-2Gal. Structurally both UEA-I and GS-IV are similar to other leguminous lectins, including members of the ConA-like lectin/glucanse superfamily, possessing a conventional legume lectin fold that comprises three β-sheets, a six-stranded back sheet, a seven- stranded front sheet, and a five-stranded S sheet which connects the front and back sheets^24,25^. Although leguminous lectins are structurally similar, their carbohydrate binding specificities and binding site location can vary widely. In the case of UEA-I and GS-IV even though the binding sites are found at different locations of the protein there is significant homology between the binding site residues (Fig. 3A). Residues involved in carbohydrate binding in the GS-IV lectin are Arg48, Ser49, Asp89, Tyr105, Gly106, Gly107, Phe108, His114, Asn135, Trp138, Tyr223^25^, and in UEA-I the binding site is a depression made up of residues Glu44, Thr86, Asp87, Gly104-Gly105, Ile130, Val134, Asn135, Trp137, Tyr220, and Arg223. Structure-based alignment of YesU with GS-IV (Fig. 3) followed by Le^x^ docking analysis using Vina-Carb (Fig. 4A) revealed a complex structure model for YesU and Le^x^ with binding affinity of −4.3 kcal/mol. Le^x^ was located within a binding pocket comprising residues Leu74, Tyr101, Pro102, His105, Arg118, Tyr121, Glu124, Arg133, Phe138 and Met202 with 5/11 residues in common with GS-IV binding residues. An additional sequence-based carbohydrate-binding site prediction technique SPRINT-CBH further supported the binding site predicted by Vina-Carb. Based on the complex structure models of YesU and Le^x^ shown in Fig. 4, we propose that Tyr101, Pro102, Arg133 and Phe138 stabilize the GlcNAc moiety, while Leu74, Tyr101, His105, Tyr121, Glu124 and Met202 are involved in Fuc binding. The stabilization of the GlcNAc moiety by Tyr101, Pro102, Arg133 and Phe138 more than likely accounts for the observed weak but significant binding to LnNT (1 H, Galβ1-4GlcNAcβ1-3Galβ1-4Glc) and (GlcNAc)_6_ (4D) observed by glycan array and SPR analyses. Further experimental studies are needed to unequivocally validate the proposed binding sites.

Similar to the leguminous lectins GS-IV and UEA-I, AAA and SP2159 are comprised of two main β-sheets and four-five anti-parallel β-strands, two forming a shallow positively charged pocket that forms the Fuc-binding pocket^30,36^. The Fuc-binding pockets in AAA and SP2159 have been attributed to hydrogen bonding with basic residues and Van der Waals contact with hydrophobic residues in the proposed binding pocket^36^. The ability of some GAGs and GAG fragments to interact with YesU may be due to the anionic sulfate interacting with YesU positively charged residues in the proposed Le^x^ binding pocket.

Given our finding that YesU from *B. subtilis* preferentially binds fucosylated glycans such as Le^x^ it is possible that YesU may be involved in the adhesion of *B. subtilis* to the gastrointestinal tract of mammals. Fucosylated glycans, particularly Lewis blood group antigens have been proposed to play a functional role in the colonisation and virulence of a number of gastrointestinal pathogens, including *Helicobacter pylori*^37^, *Campylobacter jejuni*^38^, *Salmonella enterica* sv. Typhimurium^39^, and *Pseudomonas aeruginosa*^40^. *P. aeruginosa* is particularly interesting as one of its two soluble lectins, PA-IIL binds host cell Lewis eptiopes^41^ and as we have shown here is structurally similar to YesU (Table 2 and Fig. 3). In addition, even though PA-IIL is abundantly present on the bacteria outer membrane^40^, it does not possess a signal sequence/peptide that predicts subcellular localization as determined using SignalP 4.1 (Gram-negative organism group)^42^. Similarly, YesU also does not possess a signal sequence/peptide as determined using SignalP 4.1 (Gram-positive organism group), suggesting that PA-IIL and YesU are secreted in a signal sequence/Sec independent process. Although it is unclear exactly how YesU is secreted in *B. subtilis*, evidence to support signal sequence/Sec independent secretion in Gram-positive bacteria is available. The carbohydrate binding toxin^43^, pneumolysin (Ply) from *Streptococcus pneumonia* is secreted in a signal sequence/Sec independent process. This signal sequence/Sec independent pathway is also present in *B. subtilis* as Ply knock-in mutants can also export Ply in a signal sequence/autolysis independent manner^44^. It is therefore possible that the export of YesU utilises this signal peptide/Sec independent process in *B. subtilis*.

The ability of a range of fucosylated glycans including BGBT, Le^x^, and Le^y^, but not the high Man-type glycan, Manα1-3(Manα1-3(Manα1-6)Manα1-6)Man, to inhibit the adherence of *B. subtilis* to intestinal cells (Fig. 5) provides experimental evidence to support the potential role of YesU in the colonisation of the gastrointestinal tract by *B. subtilis*. Le^x^, in particular, is a very common glycan motif in mammals, and as such represents an important receptor for pathogenic and commensal gut microbes. Although usually considered soil organisms, members of the genus Bacillus have been found to inhabit the gastrointestinal tract of insects and animals^45^. In fact there is now significant evidence that species such as *B. subtilis* should be considered gut commensals in humans rather than purely environmental microorganisms^46^. Interestingly, a homolog of YesU is also present in *B. cereus*, strains of which cause foodborne illnesses in humans^45^, but is not present in *B. thuringiensis* and *B. sphaericus*, both of which are gut commensals in insects. Insects do not express Le^x^, although they do possess a Lewis-like structure, α1,3-fucosylated *N*-acetylgalactosaminyl-β1,4-*N*-acetylglucosamine (GalNAcβ1-4(Fucα1-3)GlcNAc)^47^ that resembles Le^x^ except that a GalNAc is present rather than a Gal. The absence of a YesU homolog in Bacillus species that are insect gut commensals together with the lack of Le^x^ eptitopes in insects provides further evidence for the potential importance of YesU in the colonisation of the human gut by *B. subtilis* (and potentially *B. cereus*).

This paper validates the use of computational prediction from a method like SPOT-Struc as the first step to screen and identify potential new CBPs. We have shown that SPOT-Struc is highly predictive and ideal to uncover novel carbohydrate-binding proteins. However, prediction of the carbohydrate specificities such as reported here for YesU is more challenging for computational techniques as the sidechains rather than backbone structure play a more active role in determining lectin-binding specificity.

## Methods

### Expression and Purification of *B. subtilis* YesU Protein

The vector pMCSG68 (clone ID BSCD00606331) was obtained from DNASU plasmid repository (http://dnasu.org). The vector incorporated the *B. subtilis* hypothetical cytosolic protein 031524 (YesU; 220 residues) with a N-terminal 6xHis tag under the control of a T7 promoter. It was transformed into competent *E. coli* BL21 (DE3) cells and used for recombinant protein expression. An overnight culture of BL21 (DE3)/BSCD00606331 was used to inoculate LB broth containing ampicillin (100 µg/mL) and incubated at 37 °C with aeration. Once OD_600nm_ reached 0.4–0.6, protein expression was induced using 1 mM IPTG for a further 4 hrs and the cell pellet resuspended in binding buffer (50 mM NaHPO_4_, 300 mM NaCl; pH 8.0), lysozyme (2 mg/mL), DNaseI and protease inhibitor cocktail mix (50 µL). An additional freeze/thaw step was performed to aid in cell lysis followed by sonication and removal of the insoluble cell debris by centrifugation at 100,000 × g for 90 min. The clarified supernatant was added to 1 mL of His-select nickel affinity resin (Sigma) and rotated overnight at 4 °C using a rotational mixer. The slurry mix was then packed by gravity into 10 ml Bio-Rad chromatography column. The column was washed once with binding buffer, then washed with 50 mM NaHPO_4_, 1 M NaCl; pH 8.0 and the bound His-tagged protein eluted with 50 mM NaHPO_4_, 300 mM NaCl, 500 mM imidazole; pH 8.0 in 1 mL volume. Imidazole was removed from the sample by dialysis in PBS overnight at 4 °C. Purity was confirmed by SDS-PAGE using 12% SDS-polyacrylamide gels and Western Blot using a mouse monoclonal anti-His_6_ (1:10,000 dilution, Cell Signaling Technologies) and a goat anti-mouse horseradish peroxidase conjugated (1:10,000 dilution, Bio-Rad Laboratories) as the primary and secondary antibodies, respectively.

### Glycan Array Analysis

Glycan arrays consisting of 367 diverse glycans with and without the presence of one of three spacers (sp2, sp3 or sp4^20,48,49^) were prepared from two previously described glycan libraries^20,48,49^). Amine containing glycans with spacer’s sp2, sp3 or sp4 were synthesised as previously described^20^ and glycans without spacers were amine functionalised as previously published^50^. All glycans were suspended in 1:1 DMF:DMSO at a concentration of 500 μM and were printed onto SuperEpoxy 2 glass slides (ArrayIt, Sunnyvale, CA) using a ArrayIt SpotBot Extreme array spotter in a six pin subarray print per glass slide format. All glycans were printed in replicates of four, including four FITC control spots, per subarray using 946MP4 pins and a contact time of 1 second at 60% relative humidity, with pins being reloaded after every 12 spots.

The arrays were printed, and subsequently neutralized in 1:1 ethanolamine:DMF, followed by blocking with 0.1% BSA in 50 mM phosphate buffered saline (PBS), pH 7.4 for 5 min at 22 °C. After washing with PBS, each slide was dried by placing them in an empty 50 mL tube and centrifuging for 5 min at 200 x g. Recombinant YesU (2 µg) was incubated at a molar ratio of 1:2:4 with anti His-tag mouse monoclonal antibody (10 mg/mL, Cell Signalling Technology), anti-mouse-IgG-Alexa555 conjugated rabbit polyclonal antibody (2 mg/mL, Life Technologies) and goat conjugated anti-rabbit-IgG-Alexa555 polyclonal antibody (2 mg/mL, Life Technologies) in 50 mM Array PBS (PBS with 1.8 mM MgCl_2_ and 1.8 mM CaCl_2_), pH 7.4 containing 0.1% BSA for 15 min on ice protected from light. All subarrays on the slide were isolated using a Gene Frame (1.5 × 1.6 cm, 65 µL, Abgene, Epsom, UK) prior to the addition of the YesU-antibody mix to the array. A coverslip was applied to the GeneFrame and array slides incubated in a humidified incubator for 20 min at 22 °C in the dark. The GeneFrame and coverslip were subsequently removed and the slide gently washed twice with 50 mM PBS, pH 7.4 containing 0.001% TWEEN^®^ 20, and twice with 50 mM PBS, pH 7.4. Slides were dried by centrifugation for 5 min at 200 x g and allowed to air dried for a further 5 min.

### Fluorescent Image Acquisition and Data Processing

Fluorescence intensities of the array spots were measured using the ProScanArray microarray reader (Perkin Elmer, Waltham, MA) using the Helium-Neon 543 green excitation laser set to the Alexa Fluor 555 setting (555 nm excitation and 580 nm emission). Image analysis was carried out using the inbuilt ProScanArray imaging software, ScanArray Express (Perkin Elmer). Raw glycan signals were exported into Microsoft EXCEL. The mean background was calculated from DMF/DMSO blanks on the array plus three standard deviations. This was subtracted from each glycan to generate an adjusted signal. A one tailed T-test was performed with significance set at p = 0.05. T-test and fold change were generated using Microsoft EXCEL.

### Surface Plasmon Resonance Detection

Surface plasmon resonance (SPR) experiments were performed using a BIAcore T100 biosensor system (GE Healthcare) at 25 °C in 10 mM PBS-MgCl_2_ (pH 7.4) at a flow rate of 30 mL/min. Purified His-YesU was diluted to 50 μg/mL in PBS (pH 7.4) and loaded on flow cell 2 (FC2) of a Ni^2+^-nitrilo-triacetic acid (NTA) Series S sensor chip with 5 min of contact time. This was repeated for FC3 and FC4 to allow for triplicates and validate the reproducibility of the SPR response. FC1 had no protein loaded and was used as a blank reference. Five-fold serial dilutions of selected glycans were prepared in 10 mM PBS (pH 7.4). The glycan dilutions were loaded onto the sensor chip and assessed using single cycle kinetics (that is, after the last injection of the dilution series, the chip was regenerated with EDTA). Subsequently, the chip was re-loaded with Ni^2+^ and His-YesU before the injection of the next glycan dilution series. The specificity of the glycan binding was recorded as the response signal difference between each YesU loaded FC and the reference FC1. A 10-min dissociation time was allowed after the addition of each concentration of analyte. SPR signals were analysed using the T100 BIAcore Evaluation software and dissociation constants (*K*_D_) determine from steady-state analysis.

### Structure-based alignment and Le^x^ docking analysis

We utilized the structural alignment of YesU (pdbid: 1oq1) over GS-IV (pdbid: 1gsl) to build a candidate complex structure with the lectin Le^b^/Le^y^ of 1gsl and identified a potential binding site. The selected binding site is supported by binding residue predictions generated by SPRINT-CBH^26^. SPRINT-CBH is an orthogonal, sequence-based method, which employs a learned, SVM model to predict carbohydrate-binding residues. The final complex structure model was generated by Vina-Carb^51^; a modified version of Autodock Vina (version 1.1.2)^52^ designed to reproduce native glycosidic torsion angle preferences. In order to identify a potential binding mode, Le^x^ was relaxed with default parameters inside a 30 Å box at the predicted binding site. We did not perform a *de novo* docking over the whole structure as docking methods were found to be less accurate than template-based methods if binding sites are not known a priori^53^.

### Caco-2 adherence and glycan inhibition assays

Adherence assays were performed using Caco-2 human intestinal cell lines essentially as previously described^54^. Briefly, cells were seeded at 10^5^ cells/well in a black wall 96-well cell culture plates in minimal essential medium (MEM) for 48–72 h prior to bacterial challenge. Caco-2 cells were monitored prior to the assay to ensure a confluent monolayer of cells and that the cells had formed tight junctions. *Bacillus subtilis ATCC6633* was fluorescently labelled with carboxyfluorescein diacetate, succinimidyl ester (CFDA-SE), and 10^7^ labelled bacteria were applied to the Caco-2 cells in the presence and absence of selected glycans (at final concentrations between 0.1 and 10 mM). Following incubation at 37 °C for 60 min, protected from light, cells were carefully washed 3 times with pre-warmed PBS and the fluorescence measured at an excitation and emission wavelength of 485 nm and 535 nm, respectively using a Infinite® 200 PRO (Tecan) fluorescence plate reader.

## Electronic supplementary material

Supplementary Information
