# Supplementary material for: YesU from Bacillus subtilis preferentially binds fucosylated glycans
Source: Sci Rep. 2018 Sep 3;8:13139. doi: 10.1038/s41598-018-31241-8 (PMC6120924; doi:10.1038/s41598-018-31241-8)
Supplement: Supplementary file 1 — Supplementary Information [file 41598_2018_31241_MOESM1_ESM.docx]

# YesU from *Bacillus subtilis* preferentially binds fucosylated glycans

Joe Tiralongo^1^*, Oren Cooper^1^, Tom Litfin^1^, Yuedong Yang^2^, Rebecca King^1^, Jian Zhan^1^, Huiying Zhao^3^, Nicolai Bovin^4^, Christopher J. Day^1^, Yaoqi Zhou^1^*

^1^ Institute for Glycomics, Griffith University, Gold Coast Campus, QLD 4222, Australia

^2^ School of Data and Computer Science, Sun Yat-Sen University, Guangzhou, People's Republic of China

^3^ Queensland Institute of Medical Research, Brisbane, Queensland, Australia.

^4^ Shemyakin Institute of Bioorganic Chemistry, Russian Academy of Sciences, Moscow, Russia

Running Title: *B. subtilis* *YesU binds fucosylated glycans*

* Corresponding authors: Joe Tiralongo, Institute for Glycomics, Griffith University, Gold Coast Campus, QLD 4222, Australia, Tel: + 61 7 5552 7029; E-mail: j.tiralongo@griffith.edu.au, or Yaoqi Zhou, Institute for Glycomics, Griffith University, Gold Coast Campus, QLD 4222, Australia, Tel: + 61 7 5552 8228; E-mail: yaoqi.zhou@griffith.edu.au

## SUPPLEMENTARY DATA

**Table S1:** Library of all glycan structures present on our glycan array

**Figure S1:** The full-length coomassie stained gel and full-length western blot of the expression and purification of *B.* *subtilis* hypothetical cytosolic protein 031524 (YesU). Lane 1: Biorad precision plus protein standard, Lane 2: uninduced culture, Lane 3: induced culture, Lane 4: slurry mix (protein lysate and resin), Lane 5 wash #1 (binding buffer), Lane 6: wash #2 (wash buffer), Lane 7: Biorad precision plus protein standard, Lane 8, 9 and 10: concentrated purified YesU protein using Amicon 3K 15 mL centrifugal filter (2 mg/mL), Lane 11: Biorad precision plus protein standard, Lane 12, 13 and 14: Western blot of purified YesU protein (220 residues) concentrated using Amicon 3K 15 mL centrifugal filter at the expected 25.2 kDa.

**Figure S2:** The native molecular mass of purified YesU was determined by size exclusion chromatography on a Sephacryl S-100 column calibrated with Conalbumin (75 kDa), Ovalbumin (43 kDa), Carbonic anhydrase (29 kDa), and Ribonuclease-A (13.7 kDa). The calculated molecular mass of the predominant form of YesU was approximately 25 kDa.

**Figure S3:** SPR sensorgrams and plot of response at equilibrium against concentration for glycans. Lex (A), LNFP III (B), BGBT (C), BGHT II (D), HDi (E), LNDFH II (F), Lea (G), MFiLNO (H), Ley (I), LNnT (J), LNnDFH I (K), (GlcNAc)6 (L), Sulfo-Lea (M), LNFP (N), and BGAT (O)

**Table S1**

**Figure S1**

**Figure S2**

**Figure S3**

**Figure S3 continued**

**Figure S3 continued**
